# A20 Is Critical for the Induction of Pam3CSK4-Tolerance in Monocytic THP-1 Cells

**DOI:** 10.1371/journal.pone.0087528

**Published:** 2014-01-28

**Authors:** Jinyue Hu, Guihua Wang, Xueting Liu, Lina Zhou, Manli Jiang, Li Yang

**Affiliations:** 1 Medical Research Center, Changsha Central Hospital, Changsha, Hunan, China; 2 Cancer Center, Changsha Central Hospital, Changsha, Hunan, China; 3 Central Laboratory, Renmin Hospital of Wuhan University, Wuhan, Hubei, China; 4 Tuberculosis Research Center, Changsha Central Hospital, Changsha, Hunan, China; Wayne State University, United States of America

## Abstract

A20 functions to terminate Toll-like receptor (TLR)-induced immune response, and play important roles in the induction of lipopolysacchride (LPS)-tolerance. However, the molecular mechanism for Pam3CSK4-tolerance is uncertain. Here we report that TLR1/2 ligand Pam3CSK4 induced tolerance in monocytic THP-1 cells. The pre-treatment of THP-1 cells with Pam3CSK4 down-regulated the induction of pro-inflammatory cytokines induced by Pam3CSK4 re-stimulation. Pam3CSK4 pre-treatment also down-regulated the signaling transduction of JNK, p38 and NF-κB induced by Pam3CSK4 re-stimulation. The activation of TLR1/2 induced a rapid and robust up-regulation of A20, suggesting that A20 may contribute to the induction of Pam3CSK4-tolerance. This hypothesis was proved by the observation that the over-expression of A20 by gene transfer down-regulated Pam3CSK4-induced inflammatory responses, and the down-regulation of A20 by RNA interference inhibited the induction of tolerance. Moreover, LPS induced a significant up-regulation of A20, which contributed to the induction of cross-tolerance between LPS and Pam3CSK4. A20 was also induced by the treatment of THP-1 cells with TNF-α and IL-1β. The pre-treatment with TNF-α and IL-1β partly down-regulated Pam3CSK4-induced activation of MAPKs. Furthermore, pharmacologic inhibition of GSK3 signaling down-regulated Pam3CSK4-induced A20 expression, up-regulated Pam3CSK4-induced inflammatory responses, and partly reversed Pam3CSK4 pre-treatment-induced tolerance, suggesting that GSK3 is involved in TLR1/2-induced tolerance by up-regulation of A20 expression. Taken together, these results indicated that A20 is a critical regulator for TLR1/2-induced pro-inflammatory responses.

## Introduction

The innate immune system forms the first line in host defense against invading microbes. The recognition of conserved pathogen-associated molecular patterns (PAMPs) by pattern recognition receptors (PRRs), including toll-like receptors (TLRs) and nucleotide-binding and oligomerization domain (NOD)-like receptors (NLRs), recruits adaptor molecules, such us myeloid differentiation factor 88 (MyD88), TIR-domain- containing adapter-inducing IFN-β (TRIF) and receptor-interacting protein kinases (RIPs), to activate mitogen-activated protein kinase (MAPK) and nuclear factor-κB (NF-κB) signal pathways, resulting in the induction of pro-inflammatory cytokines and chemokines [1]–[3]. The activation of innate immunity induces the recruitment of more leukocytes, the release of highly reactive mediators to invade the infected pathogens, and finally to keep host homeostasis [1]–[3].

The activation of innate immunity induced by PRRs is tightly regulated to avoid tissue damage via the induction of ‘self-tolerance’ or ‘cross tolerance’, which is a transient state of immune cell desensitization in response to PAMP re-stimulation after a prior exposure [4]. Among them, the ‘endotoxin tolerance’ is profoundly investigated, which has been observed both in vitro and in vivo in animal model [5] as well as in humans [6]. ‘Endotoxin tolerance’ has been reported in several diseases, including sepsis [7], [8], trauma [9] and surgery [10].

Signaling block is involved in the induction of tolerance. The activation of PRRs, such as TLR4, induces the up-regulation of negative regulatory molecules, which functions as feedback regulator to inhibit TLR-induced activation of MAPK and NF-κB, resulting in the decrease of pro-inflammatory cytokine production induced by the TLR ligand re-stimulation. IRAK-M, the inactive isoform of IL-1R-associated kinases (IRAK), has been reported to be involved in the induction of endotoxin tolerance by dampening NF-κB mediated pathway [11]–[13]. Suppressors of cytokine signaling (SOCS)-1, a negative regulatory molecule of the janus kinase (JAK)- signal transducers and activators of transcription (STAT) signal cascade, is promptly induced in macrophages upon lipopolysaccharide (LPS) stimulation, and functions as a critical down-regulating factor for LPS signal pathways [14]. A20, an ubiquitin-editing enzyme (also named tumor necrosis factor alpha-induced protein 3, TNFAIP3), has been reported to be up-regulated in endotoxin tolerance, to be associated with the impaired LPS-induced signal transduction [15], and to promote the induction of LPS tolerance [16], [17].

Glycogen synthase kinase 3-α (GSK3-α) and GSK3-β are serine-threonine kinases, initially identified as enzymes to phosphorylate glycogen synthase [18]. GSK3-α and β are broadly expressed and constitutively active in most cell types, and play important roles in the regulation of many cellular functions through their capacity to phosphorylate multiple substrates, including NF-κB, cAMP response element-binding protein (CREB), activator protein-1 (AP-1), STATs, Smads, β-catenin, and nuclear factor of activated T cells (NFAT) [19]. GSK3 is also expressed in cells of the immune response, but its roles in the induction of immune response are context-dependent. The pro-inflammatory functions for GSK3 have been reported in human peripheral blood monocytes by regulation of the balance of the production between pro- and anti-inflammatory cytokines [20]. However, anti-inflammatory functions of GSK3 have also been observed [21], [22]. Recently, GSK3 has been reported to mediate cross-tolerance between TNF-α and LPS by up-regulation of A20 in macrophages [23], indicating that GSK3 is a regulator for immune homeostasis.

In this study, we found that A20 is responsible for the induction of Pam3CSK4-tolerance in THP-1 cells. The down-expression of A20 by RNA interference inhibited the induction of tolerance. The over-expression of A20 by gene transfection inhibited the induction of pro-inflammatory cytokines. Moreover, GSK3 is involved in the induction of tolerance by regulation of A20 expression, and the inhibition of GSK3 signaling down-regulated A20, and reversed Pam3CSK4 pre-treatment-induced tolerance, suggesting that GSK3 is involved in Pam3CSK4-induced pro-inflammatory immune response.

## Materials and Methods

### Reagents and antibodies

TLR1/2 ligand Pam3CSK4, TLR2/6 ligand Peptidoglycan (PGN), TLR3 ligand polyinosinic-polycytidylic acid (Poly(I:C)), TLR5 ligand flagellin, were purchased from Invivogen (San Diego, CA, USA). Rabbit anti-human A20, ERK, β-actin, IκB-α, rabbit anti-human phosphorylated p38, ERK, JNK, were purchased from Cell Signaling Technology (Beverly, MA). TLR4 ligand lipopolysaccharide (LPS) was purchase from Sigma-Aldrich (St. Louis, MO). Scramble siRNA, human A20 siRNA, were purchased from Santa Cruz (Santa Cruz, CA). Human IL-1β, TNF-α, IL-8 ELISA kits were purchased from Jiamay Biotech. (Beijing, China). GSK3 selective inhibitor SB216763 was purchase from Tocris (Bristol, UK). A20 expressing plasmid was purchased from GeneCopoeia (Germantown, MD).

### Cell culture

Monocytic cell line THP-1 was purchased from ATCC (Manassas, VA) and cultured in RPMI 1640 containing 10% FCS and antibiotics. All cells were cultured in a humidified atmosphere with 5% CO_2_ at 37°C.

### RT-PCR

Total RNA was extracted from 1–2×10^6^ cells using Trizol (Life Technologies, Gaithersburg, MD) according to the manufacturer's instructions. mRNA was reverse transcribed with RevertAid (MBI Fermemtas, Burlington Ontario, Canada) at 42°C for 60 min, and the resulting cDNA was subjected to PCR (94°C for 1 min followed by 20–25 cycles at 94°C for 30 sec, 60°C for 30 sec, 68°C for 90 sec and an extension cycle for 10 min at 68°C). PCR products were separated on 1.0% agarose gels and visualized with ethidium bromide. Forward and reverse primer pairs are listed (5' to 3') as follows:

β-actin-F: TCGTGCGTGACATTAAGGAGA

β-actin-R: ATACTCCTGCTTGCTGATCCA

A20-F: ATGAGGCCAAAAGGACAGAA

A20-R: ACTGAAAGCATTCGTTGCAG

GAPDH-F: AATCCCATCACCATCTTCCA

GAPDH-R: CCTGCTTCACCACCTTCTTG

IL-1β-F: TTGAAGCTGATGGCCCTAAAC

IL-1β-R: CACCAAGCTTTTTTGCTGTG

IL-8-F: TTGGCAGCCTTCCTGATTT

IL-8-R: TCAAAAACTTCTCCACAACCC

IRAK-M-F: TCAAGGAAACAGCCAATGTCA

IRAK-M-R: TGTGCAGGTAGTGAATGGCTT

MCP-1-F: TCTGTGCCTGCTGCTCATAG

MCP-1-R: GATTCTTGGGTTGTGGAGTGA

MyD88-F: ACTTGGAGATCCGGCAACT

MyD88-R: TGGAAGTCACATTCCTTGCT

SIGIRR-F: TTCACCTGCTCCATCCAGAA

SIGIRR-R: TCAGGTTCACCAAGAGGTCG

SOCS1-F: ATGGTAGCACACAACCAGGTG

SOCS1-R: TAGTGCTCCAGCAGCTCGAA

ST2-F: GAAATCGTGTGTTTGCCTCA

ST2-R: AAAAGCCTTGCTCATCCTTG

TLR1-F: GGCGAAACTTCAAACAAATCC

TLR1-R: ACCATGCGTGTACCAGACACT

TLR2-F: AAGGGAATTGGTTGCAGGAT

TLR2-R: ACAGATTACAGTTGGCCCTCT

TNF-α-F: ATCAGAGGGCCTGTACCTCAT

TNF-α-R: AGACTCGGCAAAGTCGAGATA

### Western blotting

1–2×10^6^ cells were lysed in 200 µl lysis buffer (20 mM Tris, pH 7.5, 150 mM NaCl, 1% Triton X-100, 1 mM EDTA, 1 mM sodium pyrophosphate, 1 mM β-glycerophosphate, 1 mM Na3VO4, 1 µg/ml leupeptin). The cell lysate was centrifuged at 12,000×g at 4°C for 5 min. Equivalent amounts of protein were electrophoresed on 10% SDS-PAGE gels and transferred onto Immobilon P membranes (Millipore). The membranes were blocked by incubation with 3% nonfat dry milk for 1 h at room temperature and then incubated with primary antibodies (1∶200–1000) in PBS containing 0.01% Tween 20 overnight at 4°C. After incubation with a horseradish peroxidase–conjugated secondary antibody (1∶2000), the protein bands were detected with SuperSigna Chemiluminescent Substrate Stable Peroxide Solution (Pierce) and BIOMAX-MR film (Eastman Kodak). When necessary, the membranes were stripped with Restore Western Blot Stripping Buffer (Pierce) and re-probed with antibodies against various cellular proteins.

### Plasmid transfection

Cells, cultured in six-well plates, were transfected with 1 µg plasmid containing sequence coding for the human A20 protein using Lipofectamine™ 2000 (Invitrogen) according to the manufacturer's instructions. Expression of A20 in the transfected cells was examined by western blot 48 h after transfection. For stable transfection, G418-resistant cells were selected after incubation with 800 µg/ml G418 for 3 weeks.

### siRNA transfection

siRNA against human A20 (sc-37655) and silencer negative siRNA control (sc-37007) were purchased from Santa Cruz Biotechnology (Santa Cruz, CA, USA). siRNA transfection reagent (sc-29528, Santa Cruz, CA, USA) was used to transfect siRNA into THP-1 cells according to the manufacturer's instructions. Briefly, 0.5 µg siRNA and 6 µl siRNA transfection reagent were used for each transfection (6-well plates, 0.5×10^6^ cells/well). The knockdown of A20 were analyzed 48 h after siRNA transfection by western blot.

### Quantitative real time RT-PCR (qRT-PCR)

The qRT-PCR was performed as described by Sun *et al* [24]. Briefly, total RNA was isolated and reverse transcribed as above. The cDNA was amplified using TaqMan Universal PCR master mix (Applied Biosystems, Foster City, CA, USA) and an ABI Prism 7500 sequence detection system (Applied Biosystems). Amplification of the target genes was normalized using the amplification levels of glyceraldehyde-3-phosphate dehydrogenase (*GAPDH*) as an endogenous control. The efficiency of the PCR was tested by amplification of the target from serially diluted cDNA generated from the reverse transcription of a stock set of human RNA. Data analysis and calculations were performed using the 2^−ΔΔ*CT*^ comparative method, as described by the manufacturer. Gene expression is shown as the fold inductions of a gene measured in TLR ligand-treated samples, relative to samples cultured with medium.

### Enzyme-linked immunosorbent assay (ELISA)

The production of IL-1β, TNF-α and IL-8 in culture supernatants was detected by enzyme-linked immunosorbent assay (ELISA) according to the manufacturers' standard protocols.

### Statistical analysis

All experiments were performed at least three times, and the representative results were shown. Results were expressed as the mean plus or minus the standard deviation (SD). Differences between groups were examined for statistical significance using Student's *t* test, and p values equal to or less than 0.05 were considered statistically significant (n = 3 for each qRT-PCR and ELISA test).

## Results

### Pam3CSK4 induced a tolerant cytokine production in THP-1 cells

**Figure 1:**
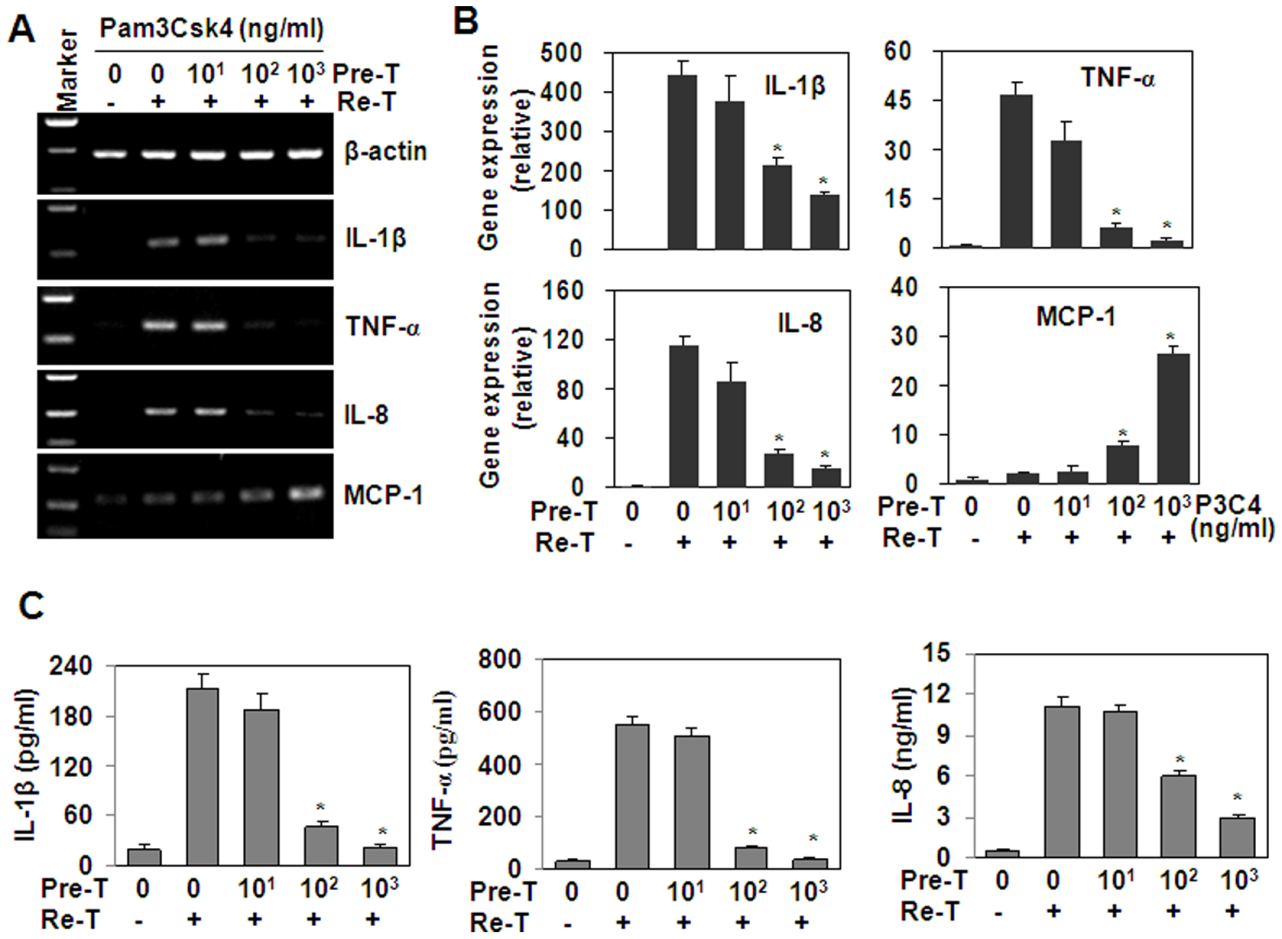
Pre-treatment (Pre-T) with Pam3CSK4 suppresses the pro-inflammatory cytokines induced by Pam3CSK4 re-treatment (Re-T). (A) Cytokine gene expression. THP-1 cells, pre-treated with the indicated concentrations of Pam3CSK4 for 6 h, were washed with PBS twice, and cultured in flesh medium for 18 h, then were re-treated with 1 µg/ml Pam3CSK4 for 1 h. The gene expression of pro-inflammatory cytokines was detected by RT-PCR. β-actin gene expression was detected as loading controls. (B) Quantitative real-time RT-PCR analysis of cytokine gene expression. THP-1 cells were treated as described as (A). The gene expression of cytokines was detected by qRT-PCR. * *P*<0.05 compared with the non-pre-treated groups. (C) ELISA analysis of cytokine protein expression. THP-1 cells, pre-treated with the indicated concentrations of Pam3CSK4 for 6 h, were washed with PBS twice, and cultured in flesh medium for 18 h, then were re-treated with 1 µg/ml Pam3CSK4 for 24 h. Cytokine proteins in the supernatant were detected by ELISA. * *P*<0.05 compared with the non-pre-treated groups.

We detected the effect of Pam3CSK4 pre-treatment on the induction of cytokines induced by Pam3CSK4 re-stimulation. The RT-PCR results showed that the pre-treatment with 100 – 1000 ng/ml Pam3CSK4 down-regulated the production of cytokines, including IL-1β, TNF-α and IL-8, induced by the re-stimulation with 1000 ng/ml Pam3CSK4 (Figure 1A). On the contrary, the production of MCP-1 was up-regulated by Pam3CSK4 pre-treatment (Figure 1A and 1B). Quantitative real time RT-PCR and ELISA results showed that the down-regulation of IL-1β, TNF-α and IL-8 was significant (Figure 1B and 1C).

### Pam3CSK4 pre-treatment down-regulated MAPK and NF-κB signaling

**Figure 2:**
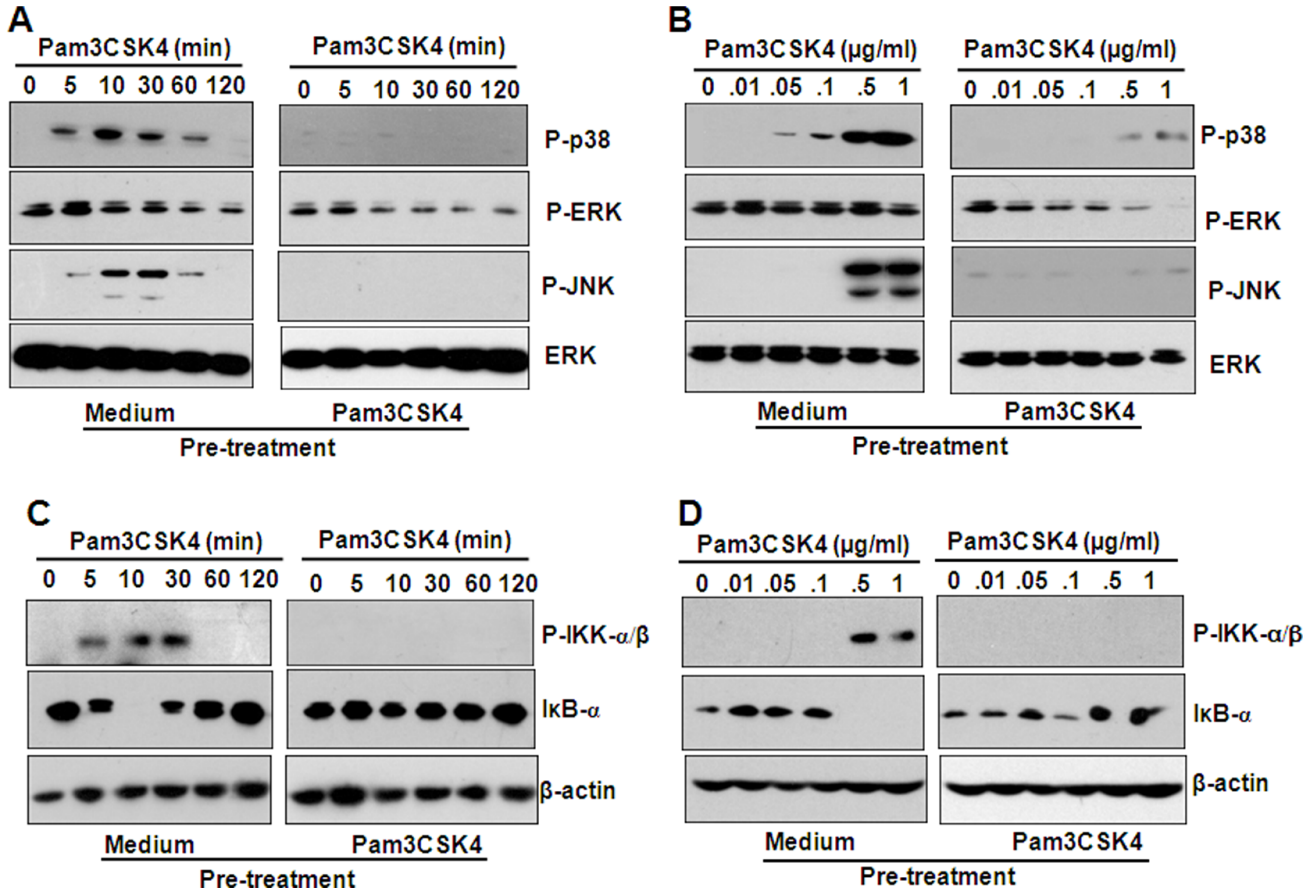
The pre-treatment with Pam3CSK4 down-regulates the activation of MAPKs and NK-κB induced by the re-treatment with Pam3CSK4. (A, B) MAPK phosphorylation. THP-1 cells, pre-treated with 1 µg/ml Pam3CSK4 for 6 h, were washed with PBS twice, and cultured with flesh medium for 18 h, then were re-treated with 100 ng/ml Pam3CSK4 for the indicated time periods (A) or with the indicated concentrations of Pam3CSK4 for 30 min (B). The phosphorylation of MAPKs was detected by western blot. ERK proteins were detected as loading controls. (C, D) IKK-α/β, IκB-α activation. THP-1 cells were treated as described as (A) (C), or (B) (D). The expression of phosphorylated IKK-α/β, and the total IκB-α were detected by western blot. β-actin proteins were detected as loading controls.

Activation of the MAPKs and NF-κB is important in the production of pro-inflammatory cytokines. In endotoxin-tolerized mouse macrophages, LPS-induced signal transduction was inhibited [5]. In this study, we detected the effect of Pam3CSK4 pre-treatment on the activation of MAPKs and NF-κB induced by Pam3CSK4 re-stimulation. Western blot results showed that the pre-treatment of THP-1 cells with 1000 ng/ml Pam3CSK4 inhibited the phosphorylation of p38 and JNK induced by various concentrations of Pam3CSK4 re-stimulation (Figure 2A), or by 1000 ng/ml Pam3CSK4 re-stimulation with various treatment time periods (Figure 2B). Pam3CSK4 pre-treatment also inhibited the phosphorylation of IκB kinase (IKK)-α/β, and the degradation of inhibitor of kappa B (IκB)-α, induced by various concentration of Pam3CSK4 re-stimulation (Figure 2C), or by 1000 ng/ml Pam3CSK4 re-stimulation with various treatment time periods (Figure 2D). These results suggested that Pam3CSK4 pre-treatment down-regulated MAPK and NF-κB signaling induced by Pam3CSK4 re-stimulation.

### The effect of Pam3CSK4 pre-treatment on the production of negative regulatory molecules

**Figure 3:**
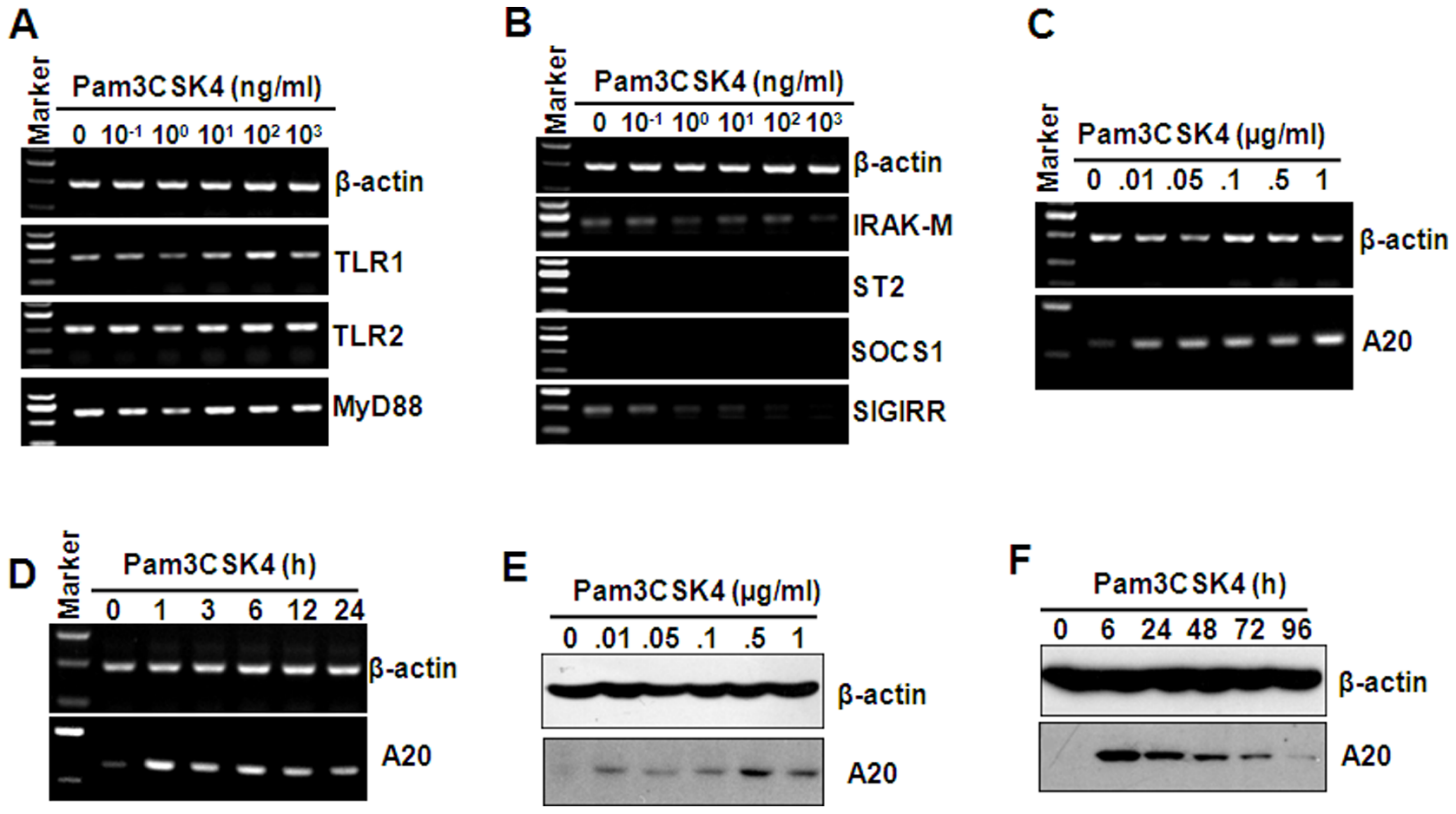
The effect of Pam3CSK4 on the expression of TLR1, 2, MyD88, and the negative regulatory molecules. (A) TLR1, 2, and MyD88 gene expression. THP-1 cells were treated with the indicated concentrations of Pam3cks4 for 24 h. The gene expression of TLR1, 2, and MyD88 was detected by RT-PCR. β-actin gene expression was detected as loading controls. (B) IRAK-M, ST2, SOCS1, SIGIRR gene expression. (C, D) A20 gene expression. THP-1 cells were treated with the indicated concentrations of Pam3CSK4 for 24 h (C) or with 1 µg/ml Pam3CSK4 for the indicated time periods (D). A20 gene expression was detected by RT-PCR. β-actin gene expression was detected as loading controls. (E, F) A20 protein expression. THP-1 cells were treated with the indicated concentrations of Pam3CSK4 for 24 h (E) or with 1 µg/ml Pam3CSK4 for the indicated time periods (F). A20 proteins were detected by western blot. β-actin proteins were detected as loading controls.

Immune system needs to constantly strike a balance between activation and inhibition to avoid tissue damage induce by inappropriate inflammatory response. Various regulatory mechanisms have been reported to tightly regulate the activation of TLRs [25]. To confirm the mechanism in Pam3CSK4-induced tolerance, the effect of Pam3CSK4 treatment on TLR1/2 and MyD88 expression was detected. RT-PCR results showed that various concentrations of Pam3CSK4 did not inhibit the expression of TLR1/2 and MyD88 (Figure 3A). Then, feedback negative regulators, including IRAK-M, SOCS1, ST2, SIGIRR, which have been reported to inhibit TLR-induced cytokine production [25], were detected by RT-PCR. The results showed that various concentrations of Pam3CSK4 did not up-regulate the expression of all this negative regulatory molecules (Figure 3B). A20, an ubiquitin-modifying enzyme, has been reported to be required for termination of Toll-like receptor responses [26]. So we detected the effect of Pam3CSK4 on the expression of A20. RT-PCR results showed that Pam3CSK4 induced significant up-regulation of A20 transcript in a dose- (Figure 3C) and time-dependent (Figure 3D) manner. Western blot results showed that Pam3CSK4 also induced significant up-regulation of A20 protein dose- (Figure 3E) and time-dependently (Figure 3F). These results suggested that A20 may play important roles in the induction of Pam3CSK4 tolerance.

### The effect of A20 on Pam3CSK4-induced tolerance

**Figure 4:**
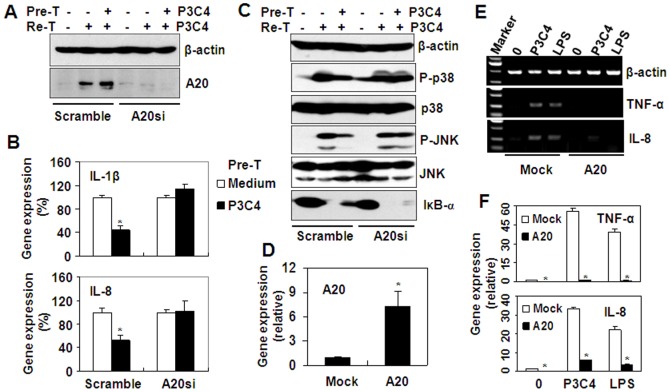
A20 is responsible for the induction of tolerance. (A) Western blot analysis of A20 expression in scramble siRNA-, or A20 siRNA-transfected THP-1 cells, pre-treated (Pre-T) with medium, or 1 µg/ml Pam3CSK4 (P3C4) for 24 h, and re-treated (Re-T) with 1 µg/ml Pam3CSK4 for 60 min. (B) Real-time RT-PCR analysis of IL-1β, and IL-8 gene expression in scramble siRNA, or A20 siRNA-transfected THP-1 cells, treated as described as (A). * *P*<0.05 compared with no pre-treatment groups. (C) Western blot analysis of p38, and JNK phosphorylation in scramble siRNA-, or A20 siRNA-transfected THP-1 cells, pre-treated with medium, or 1 µg/ml Pam3CSK4 for 24 h, and re-treated (Re-T) with 1 µg/ml Pam3CSK4 for 30 min. (D) Real-time RT-PCR analysis of A20 gene expression in mock, or A20-expressing plasmid-transfected THP-1 cells. (E) RT-PCR analysis of TNF-α, IL-1β, and IL-8 gene expression in mock, or A20-expressing plasmid-transfected THP-1 cells, treated with medium, or 1 µg/ml Pam3CSK4, or 1 µg/ml LPS for 1 h. (F) Real-time RT-PCR analysis of TNF-α, and IL-8 gene expression in mock, or A20-expressing plasmid-transfected THP-1 cells, treated as described as (E). * *P*<0.05 compared with the Mock-transfected groups.

To further prove the role of A20 in the induction of Pam3CSK4 tolerance, A20 was inhibited by siRNA transfection. Figure 4A showed that A20 expression was down-regulated by RNA interference. In the scramble siRNA-transfected control group, Pam3CSK4 induced A20 expression, but in A20 siRNA-transfected group, Pam3CSK4 did not up-regulate A20 expression (Figure 4A). Then, we detected the effect of A20 siRNA on Pam3CSK4-induced cytokine production. Quantitative real time RT-PCR results showed that in scramble siRNA-transfected group, Pam3CSK4 pre-treatment significantly down-regulated the gene expression of both IL-1β and IL-8 induced by Pam3CSK4 re-treatment (Figure 4B). However, in A20 siRNA-transfected group, the down-regulation of gene expression of both IL-1β and IL-8 was reversed (Figure 4B). Furthermore, we detected the effect of A20 RNA interference on Pam3CSK4-induced signal transduction in THP-1 cells pre-treated with or without Pam3CSK4. The results showed that in scramble siRNA-transfected group, 1 µg/ml Pam3CSK4 pre-treatment down-regulated the phosphorylation of p38 and JNK, and the degradation of IκB-α (Figure 4C), but in A20 siRNA-transfected group, the down-regulation of phosphorylation of P38 and JNK, and the degradation of IκB-α induced by Pam3CSK4 pre-treatment was reversed (Figure 4C). These results indicated that A20 is responsible for the induction of Pam3CSK4-tolerance. Subsequently, we detected the effect of A20 over-expression on Pam3CSK4-induced inflammatory responses. RT-PCR results showed that A20 transfection up-regulated the gene expression of A20 significantly (Figure 4D). In mock-transfected cells, both Pam3CSK4 and LPS induced the up-regulation of cytokines, including TNF-α and IL-8 (Figure 4E), but in A20-transfected cells, the up-regulation of cytokines induced by Pam3CSK4 and LPS was reversed (Figure 4F). These results suggested that A20 is a negative regulator for the induction of Pam3CSK4 tolerance.

### The role of A20 in the induction of cross-tolerance between Pam3CSK4 and LPS

**Figure 5:**
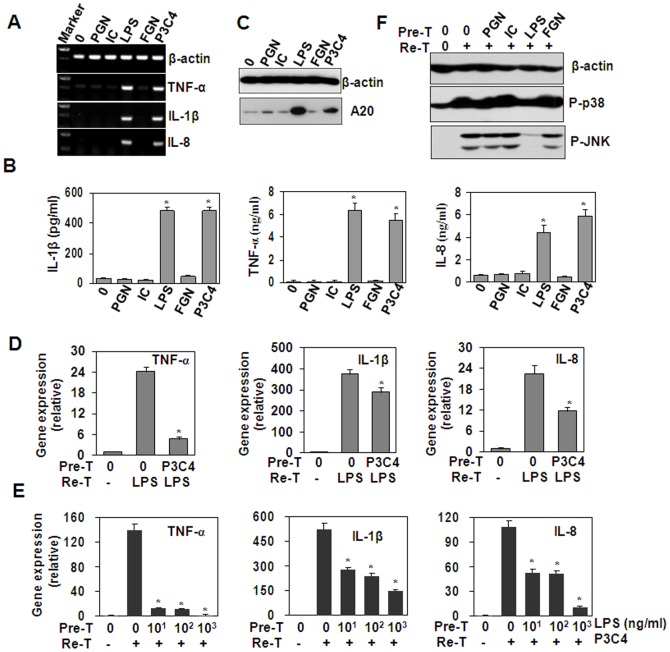
A20 is involved in the induction of cross-tolerance between LPS and Pam3CSK4. (A) The effect of various PAMPs on cytokine gene expression. THP-1 cells were treated with medium, PGN (5 µg/ml), Poly (I:C) (5 µg/ml), LPS (1 µg/ml), flagellin (FGN, 0.1 µg/ml), and Pam3CSK4 (1 µg/ml) for 1 h. The gene expression of cytokines was detected by RT-PCR. β-actin gene expression was detected as loading control. (B) The effect of various PAMPs on the cytokine protein expression. THP-1 cells were treated with indicated PAMPs for 24 h. The cytokine proteins in the supernatant were detected by ELISA. * *P*<0.05 compared with medium group. (C) The effect of various various PAMPs on A20 protein expression. THP-1 cells were treated with indicated PAMPs for 24 h. A20 protein expression was detected by Western blot. β-actin protein expression was detected as loading control. (D) The effect of Pam3CSK4 pre-treatment on LPS-induced cytokine expression. THP-1 cells, pre-treated (Pre-T) with medium, or 1 µg/ml Pam3CSK4 for 24 h, were re-treated (Re-T) with medium, or 1 µg/ml LPS for 1 h. Cytokine gene expression was detected by qRT-PCR. * *P*<0.05 compared with LPS-treated alone group. (E) The effect of LPS pre-treatment on Pam3CSK4 (P3C4)-induced cytokine expression. THP-1 cells, pre-treated (Pre-T) with the indicated concentrations of LPS for 24 h, were re-treated (Re-T) with medium, or 1 µg/ml Pam3CSK4 for 1 h. Cytokine gene expression was detected by qRT-PCR. # *P*<0.05 compared with Pam3CSK4-treated alone group. (F) The effect of PGN, Poly(I:C) (IC), LPS, flagellin (FGN) pre-treatment on Pam3CSK4 (P3C4)-induced phosphorylation of p38 and JNK. THP-1 cells, pre-treated with medium, or 10 µg/ml PGN, or 10 µg/ml Poly(I:C)(IC), or 1 µg/ml LPS, or 100 ng/ml flagellin (FGN) for 24 h, were re-treated with medium, or 1 µg/ml Pam3CSK4 for 30 min. The phosphorylation of p38 and JNK was detected by western blot. β-actin protein was detected as loading control.

In vivo LPS exposure of human blood leukocytes has been reported to induce cross-tolerance to multiple TLR ligands [6]. In this study, we detected the effect of various PAMPs on cytokine expression in THP-1 cells. RT-PCR and ELISA results showed that only LPS and Pam3CSK4 induced significant up-regulation of cytokines (Figure 5A and 5B). Western blot results showed that LPS and Pam3CSK4 up-regulated A20 protein expression (Figure 5C), suggesting that cross-tolerance may be induced between LPS and Pam3CSK4 in THP-1 cells. As expected, the pre-treatment of THP-1 cells with Pam3CSK4 down-regulated the gene expression of TNF-α, IL-1β and IL-8 induced by LPS re-stimulation (Figure 5D). On the other hand, the pre-treatment of THP-1 cells with LPS down-regulated the gene expression of TNF-α, IL-1β, IL-8 induced by Pam3CSK4 re-stimulation (Figure 5E). These results suggested that a cross-tolerance was induced between LPS and Pam3CSK4. Then, western blot was performed to detect the activation of signal transduction. The results showed the pre-treatment with LPS, but neither PGN, Poly(I:C), nor flagellin, inhibited the activation of p38 and JNK induced by Pam3CSK4 re-stimulation (Figure 5F). These results indicated that A20 is also involved in the induction of cross-tolerance between Pam3CSK4 and LPS and vice versa.

### The effect of TNF-α and IL-1β on A20 expression and Pam3CSK4-activated signaling

**Figure 6:**
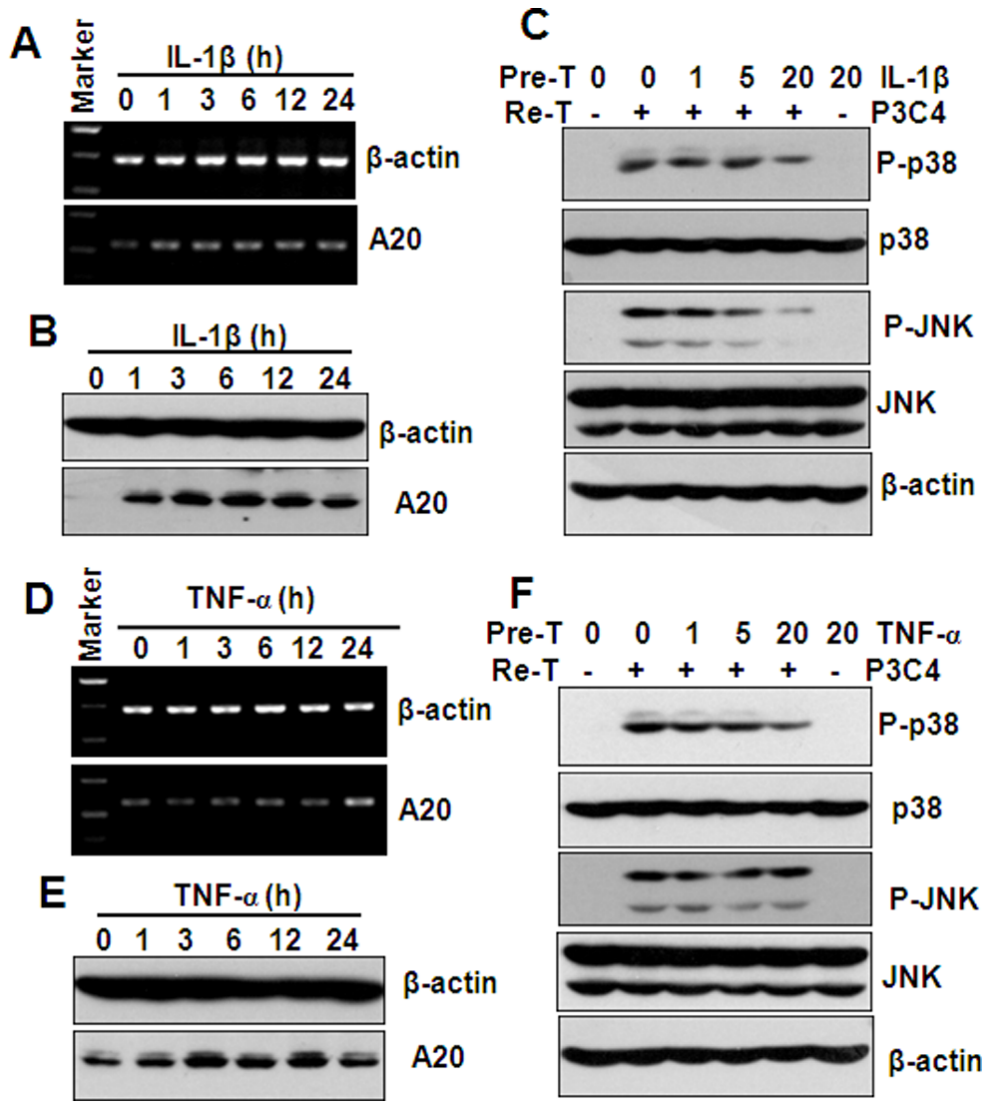
The effect of IL-1β and TNF-α pre-treatment on Pam3CSK4 (P3C4)-induced A20 expression and p38, JNK phosphorylation. (A) RT-PCR analysis of A20 gene expression in THP-1 cells, treated with 20 ng/ml IL-1β for the indicated hours. β-actin gene expression was detected as loading controls. (B) Western blot analysis of A20 protein expression in THP-1 cells, treated with 20 ng/ml IL-1β for the indicated hours. β-actin protein was detected as loading controls. (C) Western blot analysis of p38, and JNK phosphorylation in THP-1 cells, pre-treated (Pre-T) with the indicated concentrations of IL-1β for 24 h, and re-treated (Re-T) with medium, or 1 µg/ml Pam3CSK4 for 30 min. β-actin protein was detected as loading controls. (D) RT-PCR analysis of A20 gene expression in THP-1 cells, treated with 20 ng/ml TNF-α for the indicated hours. β-actin gene expression was detected as loading controls. (E) Western blot analysis of A20 protein expression in THP-1 cells, treated with 20 ng/ml TNF-α for the indicated hours. β-actin protein was detected as loading controls. (F) Western blot analysis of p38, and JNK phosphorylation in THP-1 cells, pre-treated with the indicated concentrations of TNF-α for 24 h, and re-treated with medium, or 1 µg/ml Pam3CSK4 for 30 min. β-actin protein was detected as loading controls.

TNF-α has been reported to induce cross-tolerance to endotoxin [23]. We detected the effect of IL-1β and TNF-α on the expression of A20. The results showed that IL-1β time-dependently up-regulated A20 expression at both gene and protein levels (Figure 6A and 6B). The pre-treatment of THP-1 cells with IL-1β dose-dependently inhibited the activation of p38 and JNK induced by the re-stimulation of Pam3CSK4 (Figure 6C), indicating that partial cross-tolerance may be induced between IL-1β and Pam3CSK4, and A20 may be responsible for the induction of tolerance. RT-PCR and western blot results also showed that TNF-α induced a slight up-regulation of A20 at both gene and protein levels (Figure 6D and 6E). The pre-treatment of THP-1 cells with TNF-α slightly down-regulated the activation of P38 induced by Pam3CSK4 re-stimulation (Figure 6F), suggesting that TNF-α has little effect on A20 expression and Pam3CSK4-induced signaling in THP-1 cells.

### GSK3 signal pathway is involved in the induction of Pam3CSK4 tolerance

**Figure 7:**
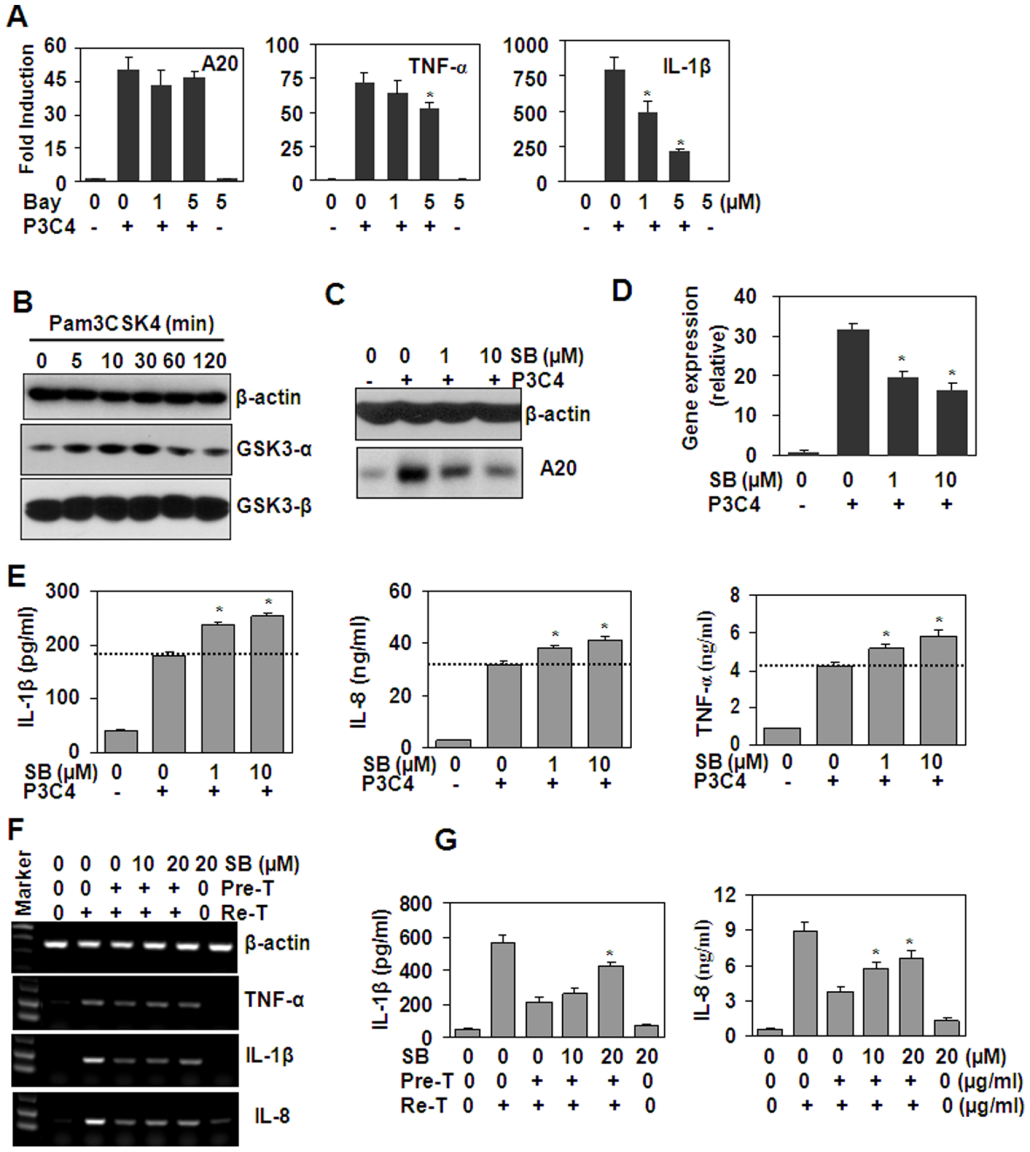
The effect of GSK3 inhibition on Pam3CSK4 (P3C4)-induced cytokine expression. (A) The effect of NF-κB inhibition on the induction of A20, TNF-α and IL-1β. THP-1 cells, pre-treated with the indicated concentrations of Bay11072 for 30 min, were re-stimulated with or without 100 ng/ml Pam3CSK4 (P3C4) for 1 h. The induction of A20, TNF-α and IL-1β was detected by qRT-PCR. * *P*<0.05 compared with the Pam3CSK4-treated alone groups. (B) The effect of Pam3CSK4 on the expression of GSK3-α and β. THP-1 cells were treated with 1 µg/ml Pam3CSK4 for the indicated time periods. The expression of GSK3-α and β was detected by western blot. β-actin protein was detected as loading control. (C) GSK3 inhibitors down-regulated Pam3CSK4-induced A20 protein expression. THP-1 cells, pre-treated with the indicated concentration of SB216763 (SB) for 1 h, were re-treated with 1 µg/ml Pam3CSK4 (P3C4) for 6 h. The protein expression of A20 was detected by western blot. β-actin protein was detected as loading controls. (D) GSK3 inhibitors down-regulated Pam3CSK4-induced A20 gene expression. THP-1 cells, pre-treated with the indicated concentration of SB for 1 h, were re-treated with 1 µg/ml Pam3CSK4 (P3C4) for 1 h. The gene expression of A20 was detected by qRT-PCR. * *P*<0.05 compared with Pam3cks4-treated alone group. (E) GSK3 inhibitors up-regulated Pam3cks4-induced cytokines. THP-1 cells, pre-treated with the indicated concentrations of SB216763 (SB) for 1 h, were re-treated with 1 µg/ml Pam3CSK4 (P3C4) for 24 h. The cytokines secreted in the supernatant were detected by ELISA. * *P*<0.05 compared with Pam3cks4-treated alone group. (F and G) GSK3 inhibition reversed Pam3CSK4 pre-treatment-induced tolerance. THP-1 cells, treated with SB216763 for 1 h, were pre-treated (Pre-T) with Pam3CSK4 (0.1 µg/ml) for 24 h, then were re-treated (Re-T) with Pam3CSK4 (0.1 µg/ml) for 1 h (F) or 24 h (G). The cytokine gene and protein expression were detected by RT-PCR (F) or ELISA (G) respectively. * *P*<0.05 compared with SB216763-non-treated but Pam3CSK4-pre-, and re-treated groups.

NF-κB has been reported to be involved in the induction of A20 transcript [27]. In this study, western blot results showed that Pam3CSK4 induced the activation of NF-κB. To determine whether NF-κB is involved in the induction of A20 in THP-1 cells, NF-κB inhibitor, BAY 11072, has been used to block NF-κB signaling. qRT-PCR results showed that NF-κB inhibition down-regulated the gene expression of TNF-α and IL-1β induced by Pam3CSK4, but did not affect the up-regulation of A20 expression (Figure 7A), suggesting that NF-κB pathway was involved in the induction of cytokines, but was not involved in the induction of A20. Recently, GSK3 pathway has been reported to mediate cross-tolerance between TNF-α and endotoxin in macrophages [23], and TLRs have been reported to activate GSK3 pathway [21], [28], [29]. So we speculated that GSK3 pathway may be involved in A20 induction. Then, we detected the effect of Pam3CSK4 on GSK3 protein expression, and the results showed that Pam3CSK4 treatment of THP-1 cells did not induce significant up-regulation of GSK3-α and β (Figure 7B). However, the inhibition of GSK3 by SB216763 down-regulated A20 expression at both protein and gene levels (Figures 7C and 7D). GSK3 inhibition also up-regulated the transcripts of cytokines, including TNF-α, IL-1β and IL-8, induced by Pam3CSK4 (Figure 7E). Moreover, SB216763 treatment of THP-1 cells reversed Pam3CSK4 pre-treatment-induced down-regulation of cytokines at both gene and protein levels (Figure 7F and 7G). These observations suggested that GSK3 was involved in the up-regulation of A20, and thus involved in the induction of Pam3CSK4 tolerance.

## Discussion

The ubiquitin-modifying enzyme A20 is required for termination of Toll-like receptor-mediated immune responses, which protected mice from endotoxic shock [26]. In A20-deficient mice, MyD88-dependent TLR signals drive the activation of T cells and myeloid cells, resulting in the premature lethality [30]. In airway epithelial cells, A20 inhibits interleukin-8 production induced by TLR2 and TLR4 [31]. A20 is also an early responding negative regulator of TLR5 signaling in intestinal epithelial cells during inflammation [32]. Leishmania donovani has been reported to modulate the TLR2-mediated pathway and the production of IL-12 and TNF-α in macrophages, which is due to the up-regulation of A20, and in vivo silencing of A20 by short hairpin RNA in BALB/c mice led to increased host-protective pro-inflammatory cytokine response and the effective parasite clearance, suggesting that Leishmania donovani might exploit host A20 to inhibit TLR2-mediated response, thus escaping the immune responses of the host [33]. Measles virus P protein has also been reported to suppress TLR signal through up-regulation of A20 [34]. In addition, A20 has been reported to be involved in the induction of LPS tolerance in mouse model [17] and in enterocytes [16]. In this study, we found that Pam3CSK4 tolerance was induced in monocytic THP-1 cells, which was also due to the up-regulation of A20. As the over-expression of A20 inhibited the production of cytokines induced by Pam3CSK4, and the down-expression of A20 inhibited the induction of tolerance induced by Pam3CSK4 re-stimulation.

Recruitment of MyD88 to TLRs mediated by PAMP stimulation plays important roles in the production of cytokines via activation of NF-κB and MAPKs [1]. The inhibition of NF-κB or MAPKs attenuates TLR-induced cytokine production [35]–[37]. We also found that inhibition of polo-like kinase attenuated TLR2- and TLR4-induced TNF-α production via suppression of MAPK and NF-κB signaling [38]. In mouse macrophages, the pre-treatment of cells with LPS inhibited the activation of MAPKs, including ERK, JNK and p38, and the activation of NF-κB induced by LPS re-stimulation [5]. LPS pre-treatment also inhibited the activation of MAPKs and NF-κB in human monocytes [39]. In our study, we found that the pre-treatment of THP-1 cells with Pam3CSK4 inhibited the phosphorylation of p38, JNK and NF-κB, suggesting that Pam3CSK4 tolerance was induced with a mechanism similar to that for LPS tolerance. As A20 is a negative regulator for MAPK and NF-κB signal transduction [33], [40], [41], we detected the effect of A20 down-regulation on the activation of p38, JNK and NF-κB, and found that A20 down-regulation reversed the inhibition of signal transduction induced by Pam3CSK4 pre-treatment. These results suggested that A20 is also involved in Pam3CSK4 pre-treatment-induced inhibition of signaling transduction.

Glycogen synthase kinase 3 is involved in TLR signaling and regulates the production of pro-inflammatory cytokines [42]. But TLR-mediated cytokine expression is differentially regulated by GSK3. On one hand, GSK3 has been found to be necessary for pro-inflammatory cytokine production following TLR stimulation [20]. GSK3 deficiency induced by pharmacological inhibitor or RNA interference down-regulated the production of pro-inflammatory cytokines, including IL-6, IL-1β, TNF-α, and IL-12p40 in human monocytes [20]. In mouse RAW264.7 macrophages, GSK3 promotes the synergistic action of interferon-γ on LPS-induced IL-6 production [43]. On the other hand, GSK3 has been reported to inhibit cytokine expression. For example, GSK3-β has been found to negatively regulate TLR4-induced IFN-β production [21]. In cardiomyocytes during LPS stimulation, GSK3-β functions to suppress TNF-α expression [22]. More recently, GSK3 has been reported to play important roles in the induction of cross-tolerance between TNF-α and LPS by up-regulation of A20 [23]. In our study, we found that GSK3 was involved in the induction of Pam3CSK4 tolerance, as GSK3 inhibition down-regulated the expression of A20 induced by Pam3CSK4, and up-regulated the expression of Pam3CSK4-induced cytokines. Moreover, GSK3 inhibition also reversed Pam3CSK4 pre-treatment-induced tolerance. These results suggested that promotion of A20 expression may be a mechanism for GSK3 to negatively regulate inflammatory response.

A20 was initially discovered as a cytokine-induced gene in human umbilical vein endothelial cells [44]. The pro-inflammatory cytokines, including TNF-α, IL-1β and bacterial LPS induce rapid, transient and robust induction of A20 transcript [44]. As the activation of NF-κB induced by cytokines or LPS has been reported to contribute to the induction of A20 [44], we detected the effect of NF-κB signaling on Pam3CSK4-induced A20 up-regulation. The results showed that NF-κB inhibition down-regulated Pam3CSK4-induced IL-1β and TNF-α, but did not regulated Pam3CSK4-induced A20 expression, suggesting that in monocytic THP-1 cells, Pam3CSK4-induced NF-κB signaling did not contribute to A20 expression. More recently, the activation of GSK3 induced by TNF-α has been found to up-regulate A20 expression. We paid our attention to the GSK3 signal pathway, and found that the inhibition of GSK3 down-regulated Pam3CSK4-induced A20 expression. However, the expression of A20 induced by Pam3CSK4 was partial down-regulated by GSK3 inhibition, the level of A20 expression induced by Pam3CSK4 was not down-regulated to the background level, suggesting that other signal pathways induced by Pam3CSK4 contribute to the induction of A20 expression.

Cross-tolerance was found between various TLR ligands [6], [45]–[47]. In our study, we found that in THP-1 cells only Pam3CSK4 and LPS induced significant up-regulation of cytokines and A20, suggesting that cross-tolerance can be induced between Pam3CSK4 and LPS. As expected, we found that both cytokine expression and MAPK signaling, induced by Pam3CSK3 or LPS, were inhibited by the pre-treatment of THP-1 cells with LPS or Pam3CSK4 respectively. These results suggested that A20 also contribute to the induction of cross-tolerance between these two TLR ligands.

Various cytokines have been reported to up-regulate A20 expression [44], suggesting that cross-tolerance can be induced between cytokines and TLR ligand [23]. In our study, we detected the effect of TNF-α and IL-1β on the expression of A20. The results showed that TNF-α treatment induced few up-regulation of A20. Accordingly, TNF-α pre-treatment induced few down-regulation of MAPK activation induced by Pam3CSK4 re-stimulation. Meanwhile, IL-1β treatment induced slight up-regulation of A20 compared with that treated by TNF-α. Accordingly, IL-1β pre-treatment induced slight down-regulation of MAPK induced by Pam3CSK4 re-stimulation. These results suggested that A20 may contribute to the induction of cross-tolerance between cytokines and Pam3CSK4 in THP-1 cell.

Beside A20, several other feedback negative regulators for TLR activation have been reported [25]. The induction of IRAK-M is essential for endotoxin tolerance in macrophages, Kupffer's cells and even in a human endotoxemia model [11]-[13]. SOCS1 is a negative regulator for macrophages activation induced by LPS [48]. ST2 functions to inhibit the signaling of IL-1 receptor and TLR4 receptor [49]. SIGIRR has also been reported to down-regulating IL-1R1 and TLR4 signaling [50]. In our study, we found that these negative regulators were not induced by TLR1/2 ligand Pam3CSK4, suggesting that they do not contribute to the induction of Pam3CSK4-tolerrance. The differences in molecular mechanism in tolerance induction may be due to the differences in cell types, TLR ligands, and human-mouse species.
